# Identifying and rationalizing the morphological, structural, and optical properties of 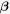-Ag_2_MoO_4_ microcrystals, and the formation process of Ag nanoparticles on their surfaces: combining experimental data and first-principles calculations

**DOI:** 10.1088/1468-6996/16/6/065002

**Published:** 2015-11-05

**Authors:** Maria T Fabbro, Carla Saliby, Larissa R Rios, Felipe A La Porta, Lourdes Gracia, Máximo S Li, Juan Andrés, Luís P S Santos, Elson Longo

**Affiliations:** 1 Department of Chemistry, CDMF, Universidade Federal de São Carlos, 13565-905, São Carlos, Brazil; 2 Department of Inorganic and Organic Chemistry, Universitat Jaume I, Campus Riu Sec, E-12071, Castellón, Spain; 3 Department of Chemistry, INCTMN, Instituto Federal do Maranhão, Monte Castelo, 65030-005, São Luís, Brazil; 4 Department of Chemistry, Universidade Tecnológica Federal do Paraná, 86036-370, Londrina, Brazil; 5 Instituto de Física de São Carlos, Universidade de São Paulo, 13560-970, São Carlos, Brazil; 6 Department of Physic and Analytical Chemistry, Universitat Jaume I, Campus Riu Sec, E-12071, Castellón, Spain; 7 CDMF, INCTMN, Instituto de Química, Universidade Estadual Paulista, Araraquara, 14801-907, Brazil

**Keywords:** theoretical calculations, morphology, electron beam irradiation, *β*-Ag_2_MoO_4_

## Abstract

We present a combined theoretical and experimental study on the morphological, structural, and optical properties of *β*-Ag_2_MoO_4_ microcrystals. *β*-Ag_2_MoO_4_ samples were prepared by a co-precipitation method. The nucleation and formation of Ag nanoparticles on *β*-Ag_2_MoO_4_ during electron beam irradiation were also analyzed as a function of electron beam dose. These events were directly monitored in real-time using *in situ* field emission scanning electron microscopy (FE-SEM). The thermodynamic equilibrium shape of the *β*-Ag_2_MoO_4_ crystals was built with low-index surfaces (001), (011), and (111) through a Wulff construction. This shape suggests that the (011) face is the dominating surface in the ideal morphology. A significant increase in the values of the surface energy for the (011) face versus those of the other surfaces was observed, which allowed us to find agreement between the experimental and theoretical morphologies. Our investigation of the different morphologies and structures of the *β*-Ag_2_MoO_4_ crystals provided insight into how the crystal morphology can be controlled so that the surface chemistry of *β*-Ag_2_MoO_4_ can be tuned for specific applications. The presence of structural disorder in the tetrahedral [MoO_4_] and octahedral [AgO_6_] clusters, the building blocks of *β*-Ag_2_MoO_4_, was used to explain the experimentally measured optical properties.

## 1. Introduction

Metal molybdates are of both theoretical and technological importance because they exhibit a wide range of electrical and optical properties that are broadly applicable in photocatalysis, solar energy conversion, and energy storage [1–14]. Recently, Zhang *et al* [15] reviewed the representative hydrothermal strategies for synthesizing inorganic semiconducting nanostructures. Several methods have been used to synthesize metal molybdates with different sizes and morphologies, including precipitation [16], sol-gel [17, 18], solid state [19], hydrothermal [20], complete evaporation of a polymer-based metal-complex precursor solution [21], and a microwave-assisted hydrothermal method [22–24].

Ag_2_MoO_4_ is an important member of the molybdate family. Several reports have been published on its attractive properties for numerous applications in materials science [25–39]. Up to now, various nano- or microstructures of Ag_2_MoO_4_ have been fabricated, such as nanoparticles [25], wire-like nanostructures [35], and flower-like microstructures [36]. Bao *et al* [37] reported the synthesis of silver molybdate nanowires decorated with Ag nanoparticles (NPs) using a solution-based chemical reaction at room temperature. Ag_2_MoO_4_ exists in two polymorphic forms: tetragonal (*α*-phase) and cubic (*β*-phase) [38–40]. Recently our research group performed a systematic first-principles investigation to calculate the structural and electronic properties of both *α*- and *β*-Ag_2_MoO_4,_ and its pressure-induced phase transitions [28]. We also investigated the correlation between theoretical calculations and our experimental data to better understand the nucleation and early evolution of Ag filaments on *β*-Ag_2_MoO_4_ crystals. These crystals were synthesized by a microwave-assisted hydrothermal method [25] and a co-precipitation method at pH 4 [29]. The electronic structures of *α*- and *β*-Ag_2_MoO_4_ have also been investigated [41].

This paper presents a detailed study, based on experimental data combined with first-principles calculations, on the correlation between growth conditions, morphology, and optical properties of *β*-Ag_2_MoO_4_. Samples were structurally characterized using x-ray diffraction (XRD) with Rietveld refinement and micro-Raman (MR) spectroscopy. The morphology, shape, and elemental composition of the crystals were verified by FE-SEM. Room temperature optical properties were investigated by ultraviolet–visible (UV–vis) absorption spectroscopy and photoluminescence (PL) measurements. According to Wulff’s law [42], the thermodynamic equilibrium morphology of the *β-*Ag_2_MoO_4_ crystals is obtained from the surface energies of the facets. The inherent instability of crystals under an electron beam means that the illuminating radiation can potentially induce atomic movement, resulting in modifications to the surfaces and internal structures. Therefore, the formation of Ag NPs on *β*-Ag_2_MoO_4_ induced by electron irradiation was studied. Electron irradiation effects must be carefully analyzed to ensure high fidelity in the characterization of particle structure. Details of this analysis are reported. These findings provide a better understanding of *β*-Ag_2_MoO_4_-based materials at the atomic scale. This new understanding will be beneficial for the fabrication of other metal oxide nanomaterials with highly exposed crystal surfaces, which have potential applications in gas sensing devices, optical devices, and catalysts.

The paper is organized as follows: section 2 describes the experimental and computational methods. In section 3, experimental and theoretical results are presented and discussed in detail. A brief summary and the main conclusions will be offered in section 4.

## 2. Experimental details

### 2.1. Synthesis of *β*-Ag_2_MoO_4_

*β*-Ag_2_MoO_4_ microcrystals were synthesized without the use of a surfactant via a co-precipitation method using ethanol as a solvent. First, 1 mmol of sodium molybdate dihydrate (Na_2_MoO_4_.2H_2_O; 99.5% purity, Sigma-Aldrich) was dissolved in 50 ml of ethanol (solution 1). Separately, 2 mmol of silver nitrate (AgNO_3_; 99.8% purity, Sigma-Aldrich) was dissolved in 50 mL of ethanol (solution 2). Solution 2 was added drop-wise to solution 1 under vigorous magnetic stirring while heating at 70 °C for 10 min. The precipitate was collected by centrifugation, washed several times with deionized water, and dried at 60 °C for 12 h.

### 2.2. Characterization

The *β*-Ag_2_MoO_4_ samples were structurally characterized by XRD using a DMax/2500 PC diffractometer (Rigaku) with Cu-K*α* radiation (*λ* = 1.5406 Å). Two different scan settings were used, both with a step of 0.02°: (1) normal routine in the 2*θ* range of 10–75° with a scanning velocity of 2° min^−1^, and (2) Rietveld routine in the 2*θ* range of 10–110° with a scanning velocity of 1° min^−1^. The phase analysis by the Rietveld method [43] was performed using the General Structure Analysis System (GSAS) software [44]. MR measurements were recorded using a Modular Raman Spectrometer (Horiba, Jobin Yvon), model RMS-550 with Ar laser excitation at 514 nm, and a fiber-microscope operating at 30–1000 cm^−1^. The morphology, microanalysis, and size of the *β*-Ag_2_MoO_4_ structures were determined by FE-SEM (Supra 35-VP, Carl Zeiss), operated at different voltages (i.e., 5, 10, 15, and 20 kV). UV–vis spectra were recorded using a Varian spectrophotometer (Model Cary 5G) in the diffuse reflection mode. PL spectra were collected with a Thermal Jarrel Ash Monospec monochromator and a Hamamatsu R446 photomultiplier. The 350.7 nm (2.57 eV) line of a krypton ion laser (Coherent Innova) was used with the output of the laser kept at 200 mW. All measurements were taken at room temperature.

### 2.3. Computational methods

First-principles total-energy calculations were carried out within the periodic density functional theory (DFT) framework using the Vienna *ab initio* Simulation Package (VASP) [45–48]. The Kohn–Sham equations were solved using the generalized gradient approximation (GGA) in the Perdew–Burke–Ernzerhof (PBE) functional to determine the electron exchange and correlation contributions to the total energy [49, 50]. The conjugate gradient (CG) energy minimization method was used to obtain the minimum (relaxed) energy state of the crystal. Atoms are considered fully relaxed when the Hellmann**−**Feynman forces converge to less than 0.005 eV Å^−1^ per atom. The electron–ion interaction was described within a plane wave basis set by the projector augmented wave (PAW) method [51]. The plane-wave expansion was truncated at the 460 eV cut-off energy. The Brillouin zones were sampled through Monkhorst–Pack special *k*-point grids that assured geometrical and energetic convergence for the *β*-Ag_2_MoO_4_ structures. Vibrational-frequency calculations were performed at a point in the harmonic approximation. The dynamical matrix was computed by numerical evaluation of the first derivative of the analytical atomic gradients.

To confirm the convergence of the total energy with respect to the slab thickness of the different surface models, the *E*_surf_ for several low-index planes was calculated. *E*_surf_ is defined as the total energy per repeating cell on the slab (*E*_slab_) minus the total energy of the perfect crystal per molecular unit (*E*_bulk_) multiplied by the number of molecular units on the surface (*n*), divided by the surface area per repeating cell of the two sides of the slab, as shown in equation (1):

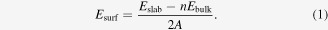



The equilibrium shape of a crystal can be calculated by using the classic Wulff construction that minimizes the total surface free energy at a fixed volume. It provides a simple relationship between the surface energy, *E*_surf_, of the (*hkl*) plane and its distance *rhkl* in the normal direction from the center of the crystallite.

The conventional cubic unit cell of *β*-Ag_2_MoO_4_ contains 8 formula units. Mo atoms occupy 8a tetrahedral sites, Ag atoms reside at the octahedral 16d position, and O atoms stay at 32e positions. The (001), (011) and (111) surfaces of *β*-Ag_2_MoO_4_ were characterized by an unreconstructed slab model using a calculated equilibrium geometry (*a* = 9.454 Å, *u*(*O*) = 0.2351) using a (3 × 3 × 1) Monkhorst–Pack special *k*-point grid. A vacuum spacer of 15 Å was introduced in the *z*-direction so that the surfaces would not interact with each other.

## 3. Results and discussion

**Figure 1.**
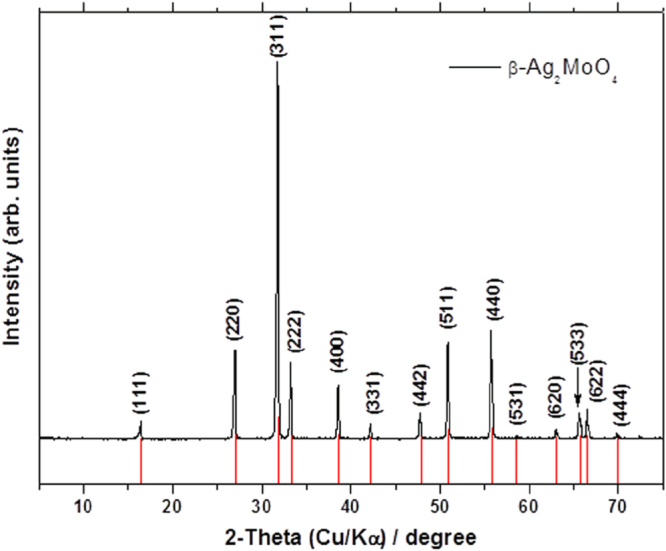
XRD patterns of the *β*-Ag_2_MoO_4_ crystals. Red lines are drawn from the ICSD card no. 28891.

XRD patterns of *β*-Ag_2_MoO_4_ are illustrated in figure 1. All XRD peaks of *β*-Ag_2_MoO_4_ crystals indicate that this material exhibits a cubic spinel structure without any deleterious phases, in good agreement with the Inorganic Crystal Structure Database (ICSD) card no. 28891 [40]. These microcrystals have sharp, well-defined diffraction peaks, which indicate a long-range structural order and crystallinity.

**Figure 2.**
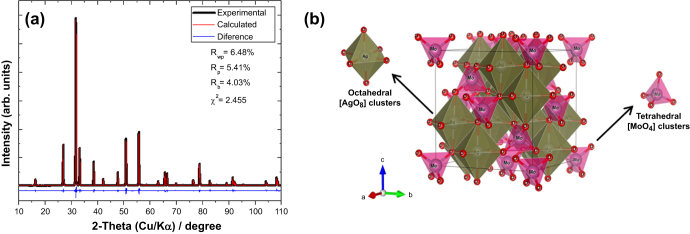
(a) Observed (black line), calculated (red line), and difference (blue line) profiles obtained after Rietveld refinement for *β*-Ag_2_MoO_4_. (b) Illustration of the *β*-Ag_2_MoO_4_ spinel structure.

The Rietveld refinement method was employed in this study (see figure 2(a)), in order to understand the crystal structure of *β*-Ag_2_MoO_4_, and prove that the as-synthesized compounds are pure single-phase crystals. Figure 2(a) shows the Rietveld refinement plots for the observed XRD patterns versus the calculated patterns of the *β*-Ag_2_MoO_4_ crystals. In particular, the fitting parameters (*R*_WP_, *R*_p_, *R*_exp_, and *χ*^2^) in figure 2(a) (inset) indicate good agreement between the refined and observed XRD patterns. These parameters confirm the existence of a single phase with a spinel structure belonging to the space group Fm3m. We compared the experimental structural parameters determined for the as-synthesized *β*-Ag_2_MoO_4_ crystals using the Rietveld refined parameters with the theoretical data obtained using first-principles calculations. The experimental values for the cell dimensions of the *β*-Ag_2_MoO_4_ crystals are 9.314 Å, while the calculations yielded a lattice parameter of 9.427 Å, which is only 1.2% greater than the experimental value. These results are consistent with the experimental values reported by Arora *et al* [38]. Arora’s group performed powder XRD measurements at ambient pressure, obtaining a single-phase material with a lattice parameter of 9.313 Å. Based on this structural analysis, we can determine the differences between the values of the angles between the [AgO_6_] and [MoO_4_] clusters, and the atomic positions.

Figure 2(b) shows a schematic representation of the *β*-Ag_2_MoO_4_ unit cells. The experimental values for the lattice parameters and atomic positions were calculated using the Visualization for Electronic and Structural Analysis (VESTA) program (Version 3.1.3 for Windows) [52], so that we could model the cubic *β*-Ag_2_MoO_4_ spinel structure. *β*-Ag_2_MoO_4_ belongs to the space group *Fd-3m*, where each Mo atom is surrounded by four O atoms, forming distorted tetrahedral [MoO_4_] clusters, whereas Ag atoms occupy the center of distorted octahedral [AgO_6_] clusters. These results indicate that the tetrahedral [MoO_4_] and octahedral [AgO_6_] clusters have lattice distortions and exhibit specific characteristics related to differences in the [O–Mo–O] and [O-Ag-O] bond angles.

It is noteworthy that the presence of defects in the local structure of these materials results from a large number of factors that are strongly dependent on synthesis methods and experimental conditions (e.g., choice of precursor, modifier, solvent, temperature, pH, processing time, etc) [53–56]. Thus, the structural characteristics of these clusters may explain some specific properties of the *β*-Ag_2_MoO_4_ crystals that have a cubic spinel structure at the atomic level.

**Figure 3.**
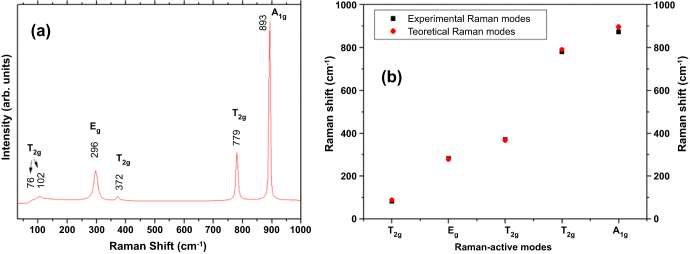
(a) Raman spectrum of *β*-Ag_2_MoO_4_. (b) A comparison of theoretical and experimental analyses of the Raman-active vibrational modes in *β*-Ag_2_MoO_4_.

MR spectroscopy was used as a probe to investigate the degree of structural order–disorder in the crystal at short-range [57]. The MR spectra of the synthesized *β*-Ag_2_MoO_4_ microcrystals are depicted in figure 3. According to group theory calculations, molybdates with a cubic spinel structure exhibit five Raman-active modes, which are represented by equation (2) [28]:

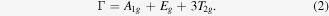



As seen in figure 3(a), the MR spectrum shows the presence of five Raman-active vibrational modes for *β*-Ag_2_MoO_4_. Figure 3(a) also shows an MR spectrum exhibiting broad vibrational modes, indicating structural disorder at short-range. The Raman-active mode observed for *β*-Ag_2_MoO_4_ at 872 cm^−1^ is ascribed to the *A*_1*g*_ mode. The symmetric stretching vibration of the Mo–O bond in [MoO_4_] clusters caused this mode. The *T*_2*g*_ mode detected at 779 cm^−1^ refers to the asymmetric stretching of this bond. We assigned a bending mode for the peak at 372 cm^−1^ in the tetrahedral [MoO_4_] cluster, whereas the *E_g_* mode at 282 cm^−1^ represents a lattice mode involving vibrations of Ag cations. Therefore, the *T*_2*g*_ modes detected at 81 cm^−1^ are also associated with the vibrations of Ag cations, as reported by Beltran *et al* [28]. Figure 3(b) confirms agreement between the observed vibrations and those reported in the literature [29]. Small shifts in observed Raman mode positions can arise from different factors such as preparation methods, average crystal size, interaction forces between the ions, or the degree of structural order in the lattice [57]. Moreover, active Raman modes confirm that *β*-Ag_2_MoO_4_ is structurally ordered at short-range, but the peaks are relatively wide, which is an indication of some structural disorder.

**Figure 4.**
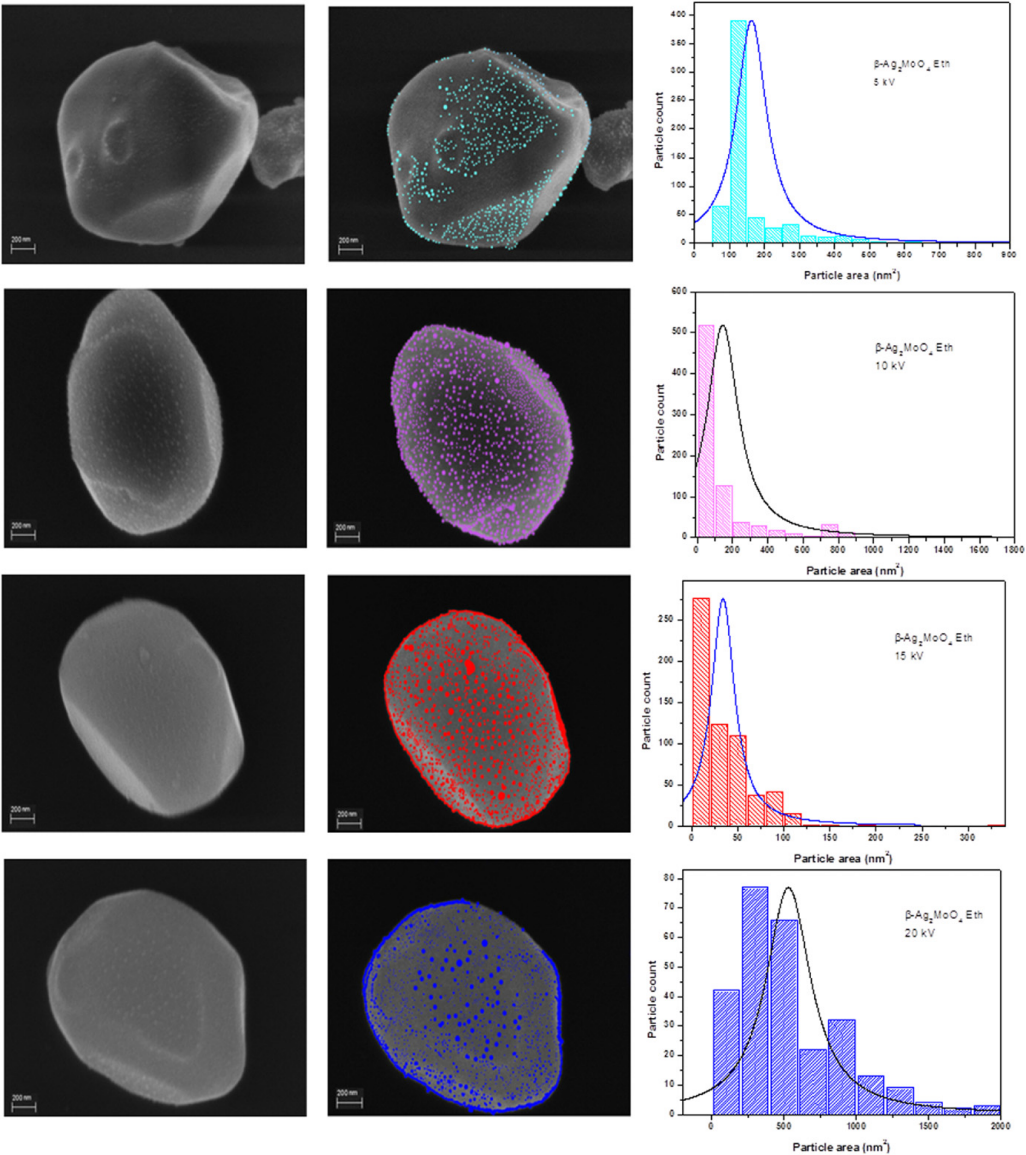
FE-SEM images of the *β*-Ag_2_MoO_4_ microcrystals (left) and growth of Ag nanoparticles on the *β*-Ag_2_MoO_4_ surface facets at 5, 10, 15, and 20 kV (right), respectively; histograms of Ag particle area distribution at different voltages after 5 min.

Our group observed the formation of Ag filaments on *β*-Ag_2_MoO_4_ via electron beam irradiation during FE-SEM imaging [41]. Such beam effects have been also reported for *α*-Ag_2_WO_4_ [58–60]. Figure 4 shows a time-resolved series of FE-SEM images obtained under high vacuum (1 × 10^−5^ Pa) during the growth of Ag nanoparticles after the absorption of energetic electrons by the *β*-Ag_2_MoO_4_ surface at 5, 10, 15, and 20 kV, respectively, and at different times: 0 and 5 min. Figure 4 also shows the size distribution of Ag nanoparticles at different voltages after 5 min of image acquisition. When the absorption of energetic electrons by the *β*-Ag_2_MoO_4_ surface occurs, the nucleation of Ag nanoparticles generating Ag vacancy defects occurs, which are related to the different average size distributions of particles. As observed in figure 4, the growth of Ag nanoparticles on the surface of *β*-Ag_2_MoO_4_ increases as the applied voltage increases. However, the growth process does not have a preferred region but rather occurs in all regions irradiated by the electron beam.

The absorption of electrons at the surface of the crystal generates an n-type semiconductor with Ag vacancies. The formation of Ag filaments caused the creation of a new p-type semiconductor, in which the nanoparticles of Ag on the *β*-Ag_2_MoO_4_ have collective oscillations of their electrons in the conduction band, which are known as localized surface plasmon resonances (LSPRs). These two systems are linked by the LSPR effect. The formation of silver filaments continues as the exposure time increases, increasing the concentration of silver vacancies. Additionally, the system that was polarized at the short- and medium-range produces a long-range polarization. This event leads to a structural rearrangement of the crystal, with the formation of new absorption centers of electrons and creation of silver filaments. It is important to note that the crystal structure is maintained along these phenomena and the formation process of Ag filaments is reversible, i.e. some Ag filaments are created on the surface while some others disappear.

The design of new complex functional materials with control of shape and surface structure is essential for a variety of technological applications and, hence, represents a fascinating challenge for researchers [61–66]. It is well known that the surface energy of facets and their stability depend on the strength of the bond between a surface atom and its nearest neighbors. Therefore, more open facets, edges, and apexes with under-coordinated atoms (i.e., reduced coordination numbers), will increase the crystal’s energy, resulting in a decreased energy barrier for atom movement. The result, then, is a crystal with lower structural stability. Conversely, high-index facets often exhibit increased chemical activity, such as catalysis, photodegradation, and antibactericide performance, as they offer active sites for adsorption of reactant molecules.

A way to control the structure and morphology of new complex functional materials would play a significant role in determining their physical and chemical properties. This ability would naturally lead to subsequent scientific and technological applications and, in particular, provide opportunities to tune and explore the optical of new structures. Nanocrystals can be grown in a variety of shapes and morphologies through the modification of surface energies of facets via adjustment of the experimental parameters, such as surfactant, and solvent choice. Therefore, control of structure and morphology is of great interest in the development of complex functional materials with customized features [67–71].

**Figure 5.**
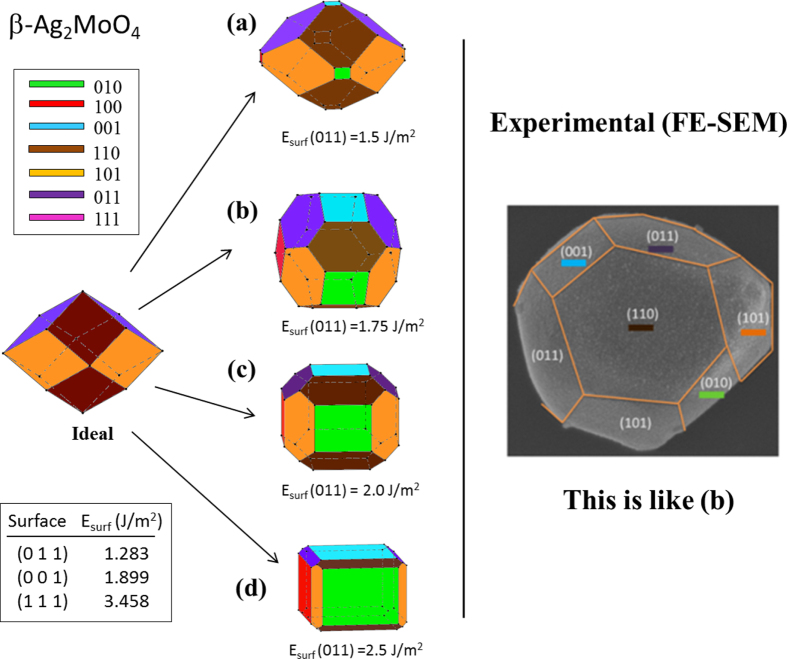
Wulff’s construction of *β*-Ag_2_MoO_4_. Morphologies and facets as a function of their surface energy values.

A range of stoichiometric surfaces for *β*-Ag_2_MoO_4_ with low Miller indices including (011), (001), and (110) surfaces were constructed and subjected to first-principles studies. The morphologies were built witha range of shapes. Figure 5 shows the Wulff construction of optimized *β*-Ag_2_MoO_4,_ and the different morphologies that could be obtained assuming different surface energy ratios for several facets. These results clearly demonstrate that the transformations between various morphologies are due to the geometric constraints imposed by the crystal structure, and are associated with the relative surface energy values of each face. This approach has the advantage that all faces are derived from the same initial crystal shape (ideal), grown as a function of their surface energy values.

**Figure 6.**
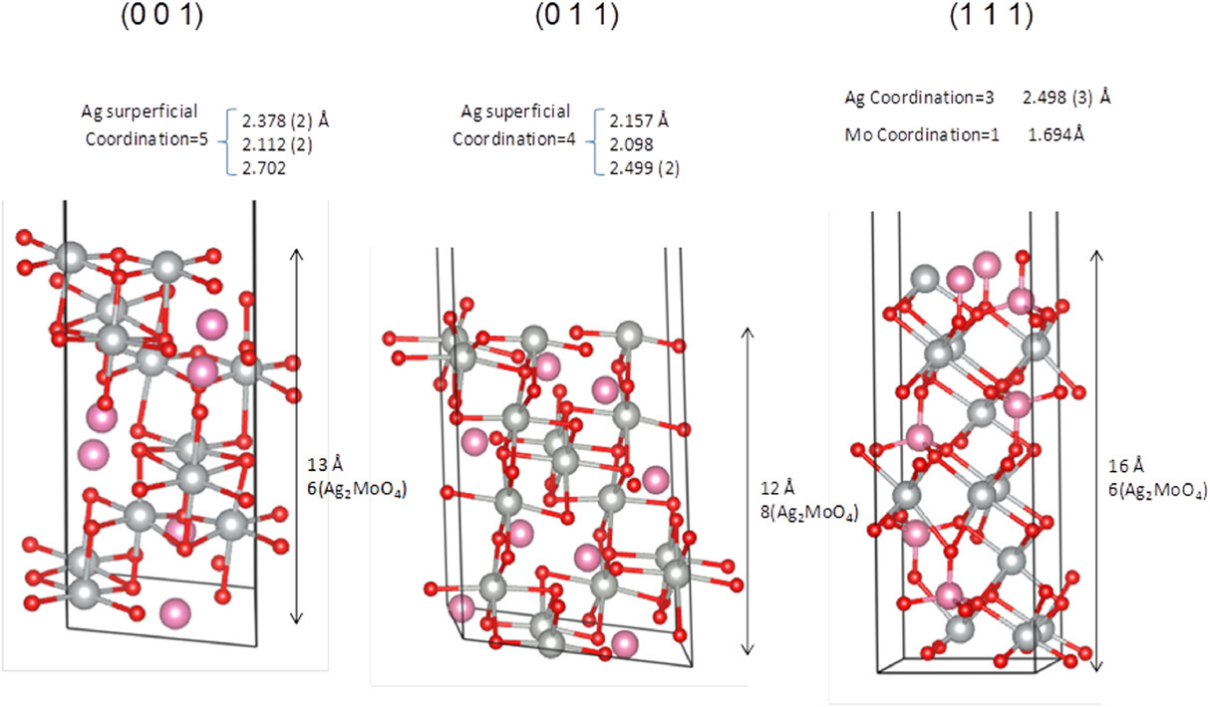
Schematic representation of the geometry for the (001), (011), and (111) faces.

It is important to note that the (011) and (001) surfaces are the most stable among all of the surfaces evaluated, whereas the (111) surface has the highest surface energy. After the corresponding convergence test on the systems, slab models containing 6, 8 and 6 molecular units for (001), (011) and (111) surfaces, with areas of 44.7, 63.2 and 38.7 Å^2^, respectively, were evaluated. It is worth noting that the (001) and (011) surfaces are O- and Ag-terminated, while the (111) surface is O- and Mo-terminated. The slab representation is shown in figure 6. In this case, the (011) orientation is the dominating surface in the predicted equilibrium shape (ideal) of the *β*-Ag_2_MoO_4_ crystals. This prediction was obtained using the Wulff construction. This analysis suggests that the Wulff model of the *β*-Ag_2_MoO_4_ crystal shapes is closely related to the surrounding chemical environment. Hence, a larger increase in the surface energy of the (011) surface rather than an increase in the other surfaces showed good agreement between the experimental and theoretical morphologies. In particular, when the values of the surface energies of both (001) and (011) surfaces are similar, experimental and theoretical morphology agrees (see figure 5(b)).

Despite some differences between the *in vacuum* conditions employed in the calculations and the actual conditions used for crystal growth in the laboratory, the most stable predicted faces usually showed the largest fraction of crystal surfaces. Therefore, the diagrams presented in figure 5 are a strong motivation to develop a relationship between crystal growth conditions and the experimentally observed morphologies. This relationship would allow us to predict morphologies resulting from different conditions. These findings promise to be very useful in interpreting changes in morphology. The above results are an illustration of how DFT calculations can provide substantial insight into the fundamental mechanisms for stabilizing micro- and nano-morphologies at the atomic level. Our work has shown that theory can be used to obtain a more in-depth understanding of experimental results. Theory may also help orient researchers in the choice of solvent, surfactants, and other relevant experimental parameters. Theory can also serve as guideline for tuning the performance of these materials by altering their shape. Using this approach, researchers can avoid following a trial-and-error process, which often follows the chemical synthesis of complex functional materials, and conduct rational structural design of new experiments.

**Figure 7.**
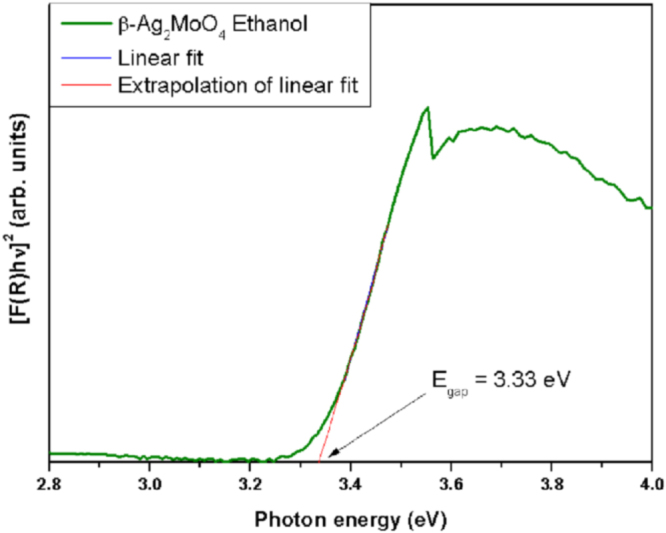
UV–visible diffuse reflectance spectrum of *β*-Ag_2_MoO_4_.

The UV–visible diffuse reflectance spectrum of *β*-Ag_2_MoO_4_ was recorded and the optical band gap energy (*E_g_*) calculated by the Kubelka and Munk equation [71], as shown in figure 7. According to Li *et al* [72], the optical band gap for *β*-Ag_2_MoO_4_ is of the indirect type. Using optical measurements, Li and his group deduced a band gap for *β*-Ag_2_MoO_4_ of 3.37 eV. In our case, the value calculated from the UV–vis spectrum is 3.33 eV. This result is in agreement with the literature [28, 29, 72] that indicates the existence of intermediary energy levels within the optical band gap of *β*-Ag_2_MoO_4_ crystals. Hence, they are formed by structural disorder of the tetrahedral [MoO_4_] and octahedral [AgO_6_] clusters that increase the numbers of electron–hole pairs.

**Figure 8.**
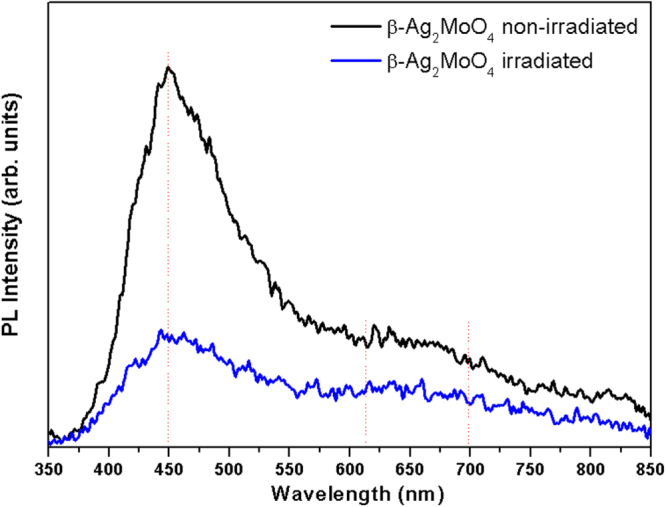
Room temperature PL spectra of the *β*-Ag_2_MoO_4_ microcrystals, excited by the 350.7 nm line of a krypton ion laser before (black) and after irradiation (blue) by an accelerated electron beam.

The PL emission spectra of both as-synthesized and irradiated *β*-Ag_2_MoO_4_ microcrystals are shown in figure 8. The PL profile suggests an emission mechanism characterized by the participation of several energy levels or light emission centers able to trap electrons within the band gap. This mechanism is typical of a multiphonon process [54]. These results indicate that the specific atomic arrangement of *β*-Ag_2_MoO_4_ microcrystals can distort the [O–Ag–O] and [O–Mo–O] bonds. Consequently, different levels of distortion on the tetrahedral [MoO_4_] and octahedral [AgO_6_] clusters in the *β*-Ag_2_MoO_4_ lattice can occur, which are mainly responsible for PL emissions. These changes can in turn alter the Ag–O and Mo–O bond lengths, the O–Ag–O and O–Mo–O bond angles and therefore the electronic and optic behavior of the material, resulting in complex coupled phenomena. This effect is similar to previous studies reported by our group on the PL emissions in tungstates [73], molibdates [74], and perovskite based materials [75–77]. Indeed, the PL emission profile analysis reveals a common feature; a pronounced maximum in the blue region (449 nm) that is related to distortions in the tetrahedral [MoO_4_] clusters [29, 78]. A weak emission was also observed in the orange (650 nm) region that indicates an ordered structure (see figure 8). On the other hand, the observations of PL emission of the irradiated *β*-Ag_2_MoO_4_ microcrystals appear to favor a nucleation process for Ag.

Therefore, electron irradiation generates structural and electronic disorder, increasing the density of electron–hole pairs. These structural changes are related to charge transfer in a crystal that contains more than one kind of cluster, and is characterized by excitations involving electronic transitions from one cluster to another. Then, clusters of a silver oxide [AgO*_x_*] absorb electron irradiation resulting in reduction of the silver ion to metallic silver (Ag°). This effect is called eletrochemireduction (ECR). In this sense, PL emission profiles are very sensitive to structural changes and serve as a powerful detector to monitor the ECR process. Therefore, the silver filaments do not form a defined interface. There is rather a combination of Ag° and 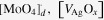 clusters. The ECR increases with time, creating clusters of silver and silver vacancies. These clusters interact, giving rise to nanowires that emerge at the surface of the crystal. Simultaneously there is a large density of 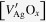 and holes (*h*^•^), which are confined within the clusters that result in the formation of new intermediate states.

## 4. Conclusions

*β*-Ag_2_MoO_4_ samples with different morphologies were synthesized by a co-precipitation method using ethanol as the solvent. *β*-Ag_2_MoO_4_ was characterized by XRD with Rietveld refinement, MR spectroscopy, and FE-SEM. Optical properties were investigated by UV–vis and PL spectroscopy at room temperature. First-principles calculations were performed to complement the experimental data. The main conclusions from this study are summarized in the following seven points.

1. The theoretical and experimental values for structural parameters and Raman vibrational frequencies are in good agreement.
2. The reversible Ag formation process during FE-SEM imaging were rationalized based on the formation of an n–p semiconductor, maintaining the structural and chemical integrity of the samples.
3. First-principles calculations were done to investigate the mechanism of the morphological transformation of surface chemistry-controlled *β*-Ag_2_MoO_4_ microcrystals. FE-SEM images also show faceted morphologies similar to the morphologies calculated by Wulff construction.
4. The combined experimental and theoretical results may provide new insights into the crystal’s plane-dependent performance.
5. The theoretical and experimental values for the band gap are in good agreement. The presence of intermediary energy levels within the optical band gap can be associated with a structural disorder of the tetrahedral [MoO_4_] and octahedral [AgO_6_] clusters, which are the building blocks of *β*-Ag_2_MoO_4_. Structural disorder enhances the presence of electron–hole pairs.
6. The PL emissions of the as-synthesized and irradiated *β*-Ag_2_MoO_4_ microcrystals depend critically on the structural disorder of tetrahedral [MoO_4_] and octahedral [AgO_6_] clusters.
7. These findings will be useful for designing and constructing novel nanostructures with specifically exposed crystal planes for surface related applications such as gas sensors, optical devices, and catalysts.
